# Update on Repetitive Transcranial Magnetic Stimulation in Post-Stroke Cognitive Rehabilitation: A Systematic Review of Randomized Clinical Trials

**DOI:** 10.3390/life16040700

**Published:** 2026-04-21

**Authors:** Davide N. Tringali, Rosario Ferlito, Rita Bella, Mariagiovanna Cantone, Rita Chiaramonte, Raffaele Ferri, Francesco Fisicaro, Michele Iacona, Maria P. Mogavero, Manuela Pennisi, Michele Vecchio, Giuseppe Lanza

**Affiliations:** 1 Department of Medical, Surgical Sciences and Advanced Technologies “G. F. Ingrassia”, University of Catania, 95123 Catania, Italy; davide.tringali02@icloud.com (D.N.T.); rbella@unict.it (R.B.); m.iacona26@gmail.com (M.I.); 2 Department of Biomedical and Biotechnological Sciences, University of Catania, 95123 Catania, Italy; ferlito.rosario@libero.it (R.F.); rita.chiaramonte@unict.it (R.C.); manuela.pennisi@unict.it (M.P.); michele.vecchio@unict.it (M.V.); 3 Neurology Unit, Policlinico University Hospital “G. Rodolico-San Marco”, 95123 Catania, Italy; m.cantone@policlinico.unict.it; 4 Disability, Territorial Rehabilitation and Prosthetic Assistance Unit, Provincial Health Authority of Catania, 95123 Catania, Italy; 5 Clinical Neurophysiology Research Unit, Oasi Research Institute-IRCCS, 94018 Troina, Italy; rferri@oasi.en.it (R.F.); paola_mogavero@libero.it (M.P.M.); 6 Primary Health Care Unit, Provincial Health Authority of Siracusa, 96100 Siracusa, Italy; 7 Department of Surgery and Medical-Surgical Specialties, University of Catania, 95123 Catania, Italy

**Keywords:** transcranial magnetic stimulation, post-stroke cognitive impairment, cognitive rehabilitation, systematic review, non-invasive brain stimulation, neural plasticity

## Abstract

**Background:** We synthesized evidence from randomized clinical trials (RCTs) published between 2019 and 2025 on repetitive transcranial magnetic stimulation (rTMS) in post-stroke cognitive impairment (PSCI) and compared different stimulation parameters, cortical targets, and combinations with rehabilitation interventions. **Methods:** A systematic review according to PRISMA guidelines examined the RCTs applying rTMS in adults with PSCI compared with control or sham groups. The primary outcome was improvement in cognitive function and functional outcomes measured with standardized scales. **Results:** Fifteen studies, involving a total of 732 patients, were included. The most frequently investigated were high-frequency (≥10 Hz) stimulation protocols of the left dorsolateral prefrontal cortex, with treatment cycles ranging from 2 to 6 weeks. Overall, rTMS was generally safe and well tolerated, with rare and mild adverse events. Several studies reported improvements in cognitive performance following rTMS, although effects were variable across trials and need caution in light of heterogeneity in stimulation protocols, sample sizes, outcome measures, and methodological quality. In most cases, rTMS or intermittent theta burst stimulation combined with structured cognitive training yielded greater cognitive and functional gains than stimulation or rehabilitation alone. This suggests a positive interaction between rTMS and cognitive training, although current evidence does not yet allow definitive conclusions. **Conclusions:** rTMS appears to be a promising strategy for post-stroke cognitive rehabilitation, particularly for attention and executive functioning. However, heterogeneity in stimulation protocols and outcome measures, along with limited sample sizes and short follow-up, reduces the certainty and comparability of current evidence. The widespread reliance on global screening tools may further underestimate domain-specific effects. Future multicentre trials with standardized protocols and more sensitive cognitive assessments are needed to clarify efficacy and guide further clinical application of rTMS in PSCI.

## 1. Introduction

Stroke remains the leading causes of long-term disability and mortality worldwide, with more than 12 million new cases reported annually, a figure that continues to rise largely due to population ageing [1]. Advances in acute stroke management have significantly improved survival; however, a substantial proportion of survivors experience persistent cognitive sequelae that critically affect functional independence, quality of life, and long-term prognosis. Post-stroke cognitive impairment (PSCI), indeed, has been reported in up to 60% of patients, representing a major clinical and socioeconomic challenge [2,3].

PSCI is a heterogeneous condition encompassing deficits across multiple cognitive domains, including attention, executive functions, memory, language, and visuospatial abilities [4]. Importantly, stroke is a strong risk factor for subsequent dementia, accelerating cognitive decline and increasing dementia risk up to fivefold compared with stroke-free populations [5]. These cognitive consequences translate into prolonged hospitalisation, higher rates of institutionalisation, caregiver burden, and healthcare costs [6].

The pathophysiology of PSCI extends beyond the focal effects of the vascular lesion. Disruption of large-scale brain networks, diaschisis phenomena, neuroinflammation, oxidative stress, and secondary neurodegenerative processes all contribute to cognitive dysfunction after stroke [7,8,9]. Moreover, clinical and biological overlaps between PSCI and neurodegenerative disorders, particularly Alzheimer’s disease (AD), have been increasingly recognised, suggesting partially shared mechanisms of cognitive vulnerability [10].

Within this context, non-invasive brain stimulation techniques have attracted growing interest as potential adjuvant tools in cognitive rehabilitation. Among them, repetitive transcranial magnetic stimulation (rTMS) is a neuromodulation technique capable of non-invasively inducing lasting changes in cortical excitability and synaptic plasticity through the delivery of focal magnetic pulses. Depending on stimulation parameters, rTMS can either enhance or inhibit neuronal activity, thereby modulating dysfunctional networks involved in cognitive processing [11,12,13]. High frequency rTMS and intermittent theta burst stimulation (iTBS) have been associated with facilitatory effects on cortical function. As known, iTBS represents a specific patterned form of rTMS, characterized by bursts of high-frequency stimulation delivered at theta rhythm, and is generally considered a time-efficient protocol with marked facilitatory effects on cortical activity [11].

In recent years, accumulating evidence supports the efficacy of rTMS in neurodegenerative and neuropsychiatric conditions, including AD and vascular cognitive impairment [14,15,16], whereas its progressively extended application to post-stroke populations has produced encouraging but not fully consistent results. Namely, variability in stimulation protocols, cortical targets, outcome measures, and follow-up duration has limited the comparability of studies and the translation of findings into clinical practice [17,18]. Although a previous meta-analysis suggests a beneficial effect of rTMS on PSCI [19], several limitations remain, including methodological heterogeneity, inclusion of outdated trials, and insufficient analysis of domain-specific cognitive outcomes. Moreover, comparative data on different stimulation paradigms and their interaction with cognitive rehabilitation are still scarce [20].

Based on this background, the present systematic review aims to provide an updated and focused synthesis of randomized controlled trials (RCTs) published between 2019 and 2025 investigating the efficacy and safety of rTMS and related protocols in adult patients with PSCI. Particular attention has been paid to stimulation parameters, cortical targets, integration with cognitive training, and effects on specific cognitive domains.

## 2. Materials and Methods

### 2.1. Inclusion/Exclusion Criteria and Selection Strategy

This systematic review was conducted in accordance with the PRISMA 2020 (Preferred Reporting Items for Systematic Reviews and Meta-Analyses) guidelines, with the aim of ensuring transparency and methodological rigour in the synthesis process.

The article search and selection phase was structured according to the PICO model:

- Population: patients with stroke (ischaemic or haemorrhagic), regardless of phase (acute, subacute, or chronic).
- Intervention: rTMS, including iTBS protocols, administered using any protocol (frequency, duration, brain target, etc.).
- Comparison: no treatment, placebo (sham TMS) or other forms of standard cognitive rehabilitation.
- Outcome: improvement in cognitive functions, assessed using standardised neuropsychological tests.

Inclusion criteria were: RCTs; publication between 2019 and 2025; patients with PSCI; intervention based on rTMS, including iTBS protocols; cognitive outcomes assessed through validated tests; articles in English with full text available for consultation. Exclusion criteria: studies not focused on PSCI (e.g., spasticity, motor/sensory deficit, pain, etc.); studies on animals or paediatric populations; case reports/series, narrative reviews, editorials/commentaries; full-text not available/not accessible; interventions other than TMS/TBS.

The article selection procedure was divided into two phases: initially, two independent reviewers (D.N.T. and M.I.) evaluated both titles and abstracts of the studies retrieved through bibliographic research; subsequently, the full text of each selected article was examined to verify its compliance with the inclusion criteria. Any discrepancy between the reviewers were resolved through discussion and consensus of a third author (G.L.), although a formal inter-rater agreement (e.g., Cohen’s κ) was not calculated for the screening process.

The entire selection process was represented using the PRISMA flow diagram. Only RCTs investigating the effects of rTMS and related paradigms of stimulation on cognitive abilities in subjects with PSCI were included.

### 2.2. Search Strategy

A systematic search was conducted in two main databases, PubMed and Cochrane Library, respectively. The search began in January 2019 and ended in June 2025. Eligible articles present in the databases during that period were therefore included. The PubMed search string was developed using a combination of MeSH terms and free-text keywords related to TMS, stroke, cognitive impairment, and rehabilitation, including synonyms and variations to improve search sensitivity while maintaining relevance to the PICO framework: ((“Transcranial Magnetic Stimulation” [Mesh] OR “transcranial magnetic stimulation” OR TMS OR rTMS OR “repetitive transcranial magnetic stimulation” OR “theta burst stimulation” OR iTBS) AND (“Stroke” [Mesh] OR stroke OR “cerebrovascular accident” OR “brain infarction” OR “post-stroke”) AND (“Cognition” [Mesh] OR cognition OR “cognitive function” OR “cognitive impairment” OR “cognitive dysfunction” OR “cognitive decline” OR “neurocognitive disorder*” OR PSCI) AND (“rehabilitation” OR “cognitive rehabilitation” OR “neurorehabilitation” OR “executive function*” OR attention OR memory) AND (randomized controlled trial [pt] OR controlled clinical trial [pt] OR random* OR trial)).

For Cochrane, the following search string was used: (*stroke” OR “cerebrovascular accident” OR “brain infarction” OR “post-stroke”) AND (“cognitive impairment” OR “cognitive dysfunction” OR “cognitive decline” OR “cognitive disorders” OR “post-stroke cognitive impairment” OR “neurocognitive disorders”) AND (“repetitive transcranial magnetic stimulation” OR “rTMS” OR “transcranial magnetic stimulation” OR “TMS” OR “intermittent theta burst stimulation” OR “iTBS”) AND (“rehabilitation” OR “cognitive rehabilitation” OR “executive function” OR “attention” OR “memory” OR “non-invasive brain stimulation”).*

To ensure methodological rigor and feasibility, the literature search was conducted using PubMed and Cochrane Library, which are widely recognized as core databases for both biomedical and clinical research, and provide high coverage of peer-reviewed RCTs and systematic reviews in this field. Moreover, while search strings were designed to balance specificity and relevance to the PICO framework, we used more extensive keyword combinations or additional synonyms in order to increase sensitivity and identified further eligible studies.

For each of the studies included in the review, information was collected on author, year of publication, pathology and population analysed, study design, sample size, characteristics of the rTMS intervention, control group, tools used for outcome assessment, follow-up duration and main results obtained.

### 2.3. Risk of Bias Assessment

The methodological quality of the studies was assessed using the Cochrane Risk of Bias 2.0 (RoB 2.0) tool, which examines five main domains: correct randomization; presence of deviations from the planned intervention; reliability in measuring outcomes; management of missing data; selection of reported outcomes. Accordingly, the risk of bias was assessed as low, moderate, or high.

In addition, the overall quality of the evidence for each outcome was assessed using the GRADE (Grading of Recommendations, Assessment, Development and Evaluation) system. This approach considers five methodological aspects: risk of bias, inconsistency between results, imprecision of estimates, indirectness of evidence and risk of publication bias. Given the heterogeneity of study designs, stimulation protocols, and outcome measures, a formal tabular GRADE summary was not considered appropriate; therefore, the certainty of evidence was synthesized narratively across outcomes.

## 3. Results

**Figure 1:**
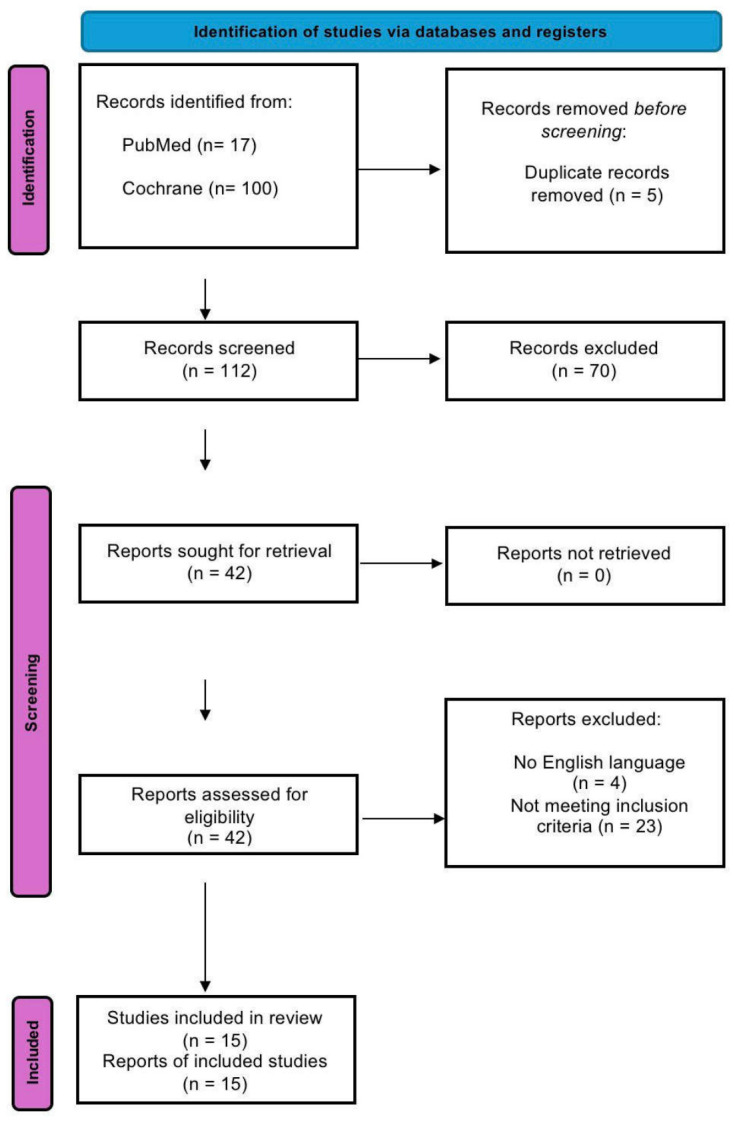
PRISMA 2020 flow diagram illustrating the study selection process.

The systematic search, based on the above-mentioned strings, identified a total of 117 articles: 17 from PubMed and 100 from the Cochrane Library. After removing one duplicate, 112 articles were screened, of which 70 were excluded based on title and abstract. The remaining 42 articles were evaluated by reading the full text. Twenty-seven of these were excluded for reasons related to the study design or irrelevant intervention. Therefore, a total of 15 RCTs were included in this review. The selection process is illustrated in the PRISMA diagram (Figure 1; Table S1), whereas Table 1 summarizes the key elements of the articles considered here.

Overall, the analysis of the RCTs indicates that rTMS was frequently associated with improvements in cognitive performance in patients with PSCI. However, these effects were not uniform across studies and varied according to stimulation parameters, cortical targets and treatment protocols, and outcome measures used. In many studies, active stimulation protocols were associated with greater gains than sham or control conditions. However, these effects were not consistent across trials and should be interpreted cautiously, given variability in study design, sample size, and risk of bias. Global cognitive screening tools, such as the Mini-Mental State Examination (MMSE) and the Montreal Cognitive Assessment (MoCA), were the most commonly used outcome measures. These were often complemented by domain-specific tests assessing attention, working memory, executive functions, and episodic memory. In most trials, cognitive improvements were already evident at the end of the stimulation cycle and, when assessed, were partially maintained at short-term follow-up.

### 3.1. Cognitive Domains Involved

Across the included studies, attentional-executive domains were among the most frequently reported areas of improvement. However, the magnitude and consistency of these effects varied across trials, and findings were not uniformly replicated. While several studies suggest potential benefits in attention, working memory, and executive functioning, the heterogeneity of study designs and outcome measures limits the extent to which these effects can be considered robust or generalizable.

### 3.2. Stimulation Protocols and Comparisons

Most trials applied high-frequency rTMS (5–10 Hz) or iTBS targeting the left DLPFC, with treatment durations ranging from 10 to 20 sessions over 2–4 weeks. These protocols were superior to sham stimulation or cognitive training alone. Comparative studies suggested that both 5 Hz rTMS and iTBS are effective in improving global cognition, with possible domain-specific differences, such as a relative advantage of 5 Hz stimulation for attention and iTBS for immediate memory.

Low-frequency stimulation of the contralesional hemisphere was less frequently investigated but appeared to confer additional benefits when combined with structured cognitive rehabilitation, supporting the relevance of interhemispheric balance and network-level modulation in PSCI recovery.

### 3.3. Integration with Cognitive Training and Durability of Effects

A recurring observation across several studies is that rTMS is often delivered in combination with structured cognitive training, and some trials report greater improvements with combined interventions compared with control conditions. However, only a minority of studies were specifically designed to directly compare combined vs. single interventions. Therefore, while the available findings suggest a potential interaction between neuromodulation and cognitive training, the evidence remains largely indirect and does not allow firm conclusions regarding a true synergistic effect.

Typically, cognitive training followed rTMS, including iTBS, sequentially, with a typical interval ≤ 30 min between stimulation and training [22,23,26,28,32,34], whereas no study used concurrent stimulation during task performance. Training protocols primarily targeted attention, executive functions, and memory, thus matching the cognitive domains most responsive to left DLPFC stimulation. Direct comparisons confirm superior efficacy of the combined approach versus single interventions [22,26], raising the hypothesis that DLPFC stimulation may facilitate subsequent training-induced plasticity. However, this interpretation remains speculative and requires confirmation in studies specifically designed to test causal mechanisms.

From a mechanistic perspective, the interaction between rTMS and cognitive training has been interpreted within frameworks of activity-dependent plasticity, including Hebbian-like mechanisms and “priming” effects. However, beyond these theoretical models, several studies included in this review provide preliminary empirical support for neurophysiological and network-level modulation associated with cognitive improvement [10,20]. This approach is coherent with the hypothesis that stimulation-induced increases in cortical excitability create a time window favourable for learning-dependent plasticity [10,20]. In contrast, protocols involving simultaneous (online) cognitive training during stimulation were not systematically investigated in RCTs, and therefore their comparative efficacy remains unclear.

Another relevant aspect concerns the potential interaction between stimulation targets and the type of cognitive training performed. Most studies applied high-frequency stimulation to the left DLPFC, a key hub for executive control and working memory, and combined it with multi-domain cognitive training programmes targeting attention, executive functions, memory, and problem-solving abilities. This convergence suggests a degree of functional alignment between the stimulated cortical region and the cognitive domains being trained. However, the available evidence does not yet allow for firm conclusions regarding task-specific or region-specific matching strategies, as few studies were explicitly designed to test whether targeting specific cortical areas enhances the efficacy of domain-specific cognitive exercises.

Overall, current findings support the concept that rTMS acts as a facilitator of experience-dependent neuroplasticity, enhancing the effects of cognitive rehabilitation when delivered in close temporal association. Nevertheless, future studies should adopt more rigorous *designs (e.g., factorial* trials) to disentangle the independent and combined effects of stimulation and training, as well as to determine the optimal timing (online vs. offline) and the most effective pairing between stimulation targets and cognitive tasks. As such, although combined interventions were frequently associated with greater cognitive and functional improvements compared with control conditions, the extent to which this reflects an additive or synergistic effect remains uncertain due to the limited number of direct comparative designs.

### 3.4. Safety and Tolerability

In all trials examined, rTMS was found to be generally safe and well tolerated in this type of patients. The rare adverse events reported (such as headache or fatigue) were mostly mild and transient, with no serious complication attributable to the treatment. This safety profile, combined with the documented benefits in the areas of attention, executive function and memory, supports the use of rTMS as a possible complementary option in post-stroke cognitive rehabilitation programmes.

### 3.5. Risk of Bias

For each study included, a systematic assessment of the risk of bias was conducted using RoB 2.0, a tool developed specifically for RCTs. For each domain, a risk assessment (low, some concern or high) was made, based on which an overall assessment of the RoB for each study was then formulated; a summary of these assessments is presented in Table 2. Overall, the risk of bias was low to moderate in most trials, with higher risk mainly confined to small pilot studies or non-sham-controlled designs.

Although the overall risk of bias was low to moderate in most included studies, this aspect has important implications for the interpretation of the results. In particular, studies with moderate or high risk of bias, often due to small sample sizes, incomplete reporting of allocation procedures, or lack of adequate blinding, may be more prone to overestimating treatment effects. From a GRADE-informed perspective, these methodological limitations contribute to reducing the overall certainty of the evidence and limit confidence in the magnitude and consistency of the observed effects. Therefore, while the available data suggest a potential benefit of rTMS on post-stroke cognitive outcomes, the strength of these conclusions remains constrained by study quality, and findings should be interpreted with appropriate caution.

As a whole, these methodological considerations were taken into account in the interpretation of results and contributed to the overall assessment of low-to-moderate certainty of evidence.

### 3.6. Heterogeneity of Stimulation Protocols and Outcome Patterns

Across the included RCTs, a marked heterogeneity was observed in stimulation parameters, cortical targets, and intervention design. Stimulation frequencies ranged from low-frequency (1 Hz) inhibitory protocols [28,34] to high-frequency (5–10 Hz) paradigms [22,23,24,29] and patterned protocols such as iTBS [26,27,32], with substantial variability in stimulation intensity, number of pulses, and duration of treatment cycles (typically 2–6 weeks). When examined descriptively, high-frequency rTMS, including iTBS, targeting the left DLPFC were the most frequently associated with improvements in global cognition, attention, and executive functions [22,23,24,25,29,31]. In contrast, low-frequency stimulation of the contralesional hemisphere was less frequently investigated but showed potential benefits, particularly when combined with structured cognitive rehabilitation [28,34]. Moreover, studies combining rTMS with structured cognitive training generally reported greater and more consistent improvements compared with those using stimulation alone or less structured interventions [22,23,24,26,30,32], although direct comparative evidence remains limited.

Despite these emerging patterns, the variability in stimulation protocols, cortical targets, outcome measures, and follow-up duration limits direct comparability across studies and suggests that the observed effects should be interpreted in relation to specific intervention characteristics rather than as a uniform class effect. Notably, neuronavigation-based targeting was applied in some recent trials [24], potentially contributing to greater precision and more consistent outcomes.

## 4. Discussion

### 4.1. Main Findings

This systematic review suggests that rTMS may represent a promising approach for the rehabilitation of post-stroke cognitive disorders. Potential benefits have been reported across multiple studies, particularly in attentional-executive domains and working memory. However, the evidence remains heterogeneous, and the consistency of these effects across different protocols and populations is limited [21,22,23,24,25,26,32,33,34]. More in details, high-frequency rTMS, including iTBS protocols, targeting the left DLPFC has been frequently investigated and is often associated with improvements in neuropsychological outcomes. These findings are consistent with the latest guidelines on the therapeutic use of rTMS [11] and with meta-analyses indicating DLPFC stimulation [19] as a potential additional option in post-stroke cognitive disorders and vascular or mixed forms of decline. Nevertheless, these associations are not uniform across studies and should be interpreted in light of methodological variability and potential sources of bias.

Although some systematic reviews and meta-analyses previously examined the role of rTMS in cognitive impairment, including post-stroke conditions, the present study provides several elements of novelty. First, this review focuses exclusively on recent RCTs, published between 2019 and 2025, thereby offering an updated synthesis that incorporates the most current methodological advances, including the increasing use of neuronavigation, iTBS, and multimodal neurophysiological and neuroimaging outcomes. Compared with earlier reviews often including heterogeneous study designs (e.g., observational studies or older trials), our approach enhances the level of evidence and clinical relevance.

Second, unlike previous reviews that primarily reported global cognitive outcomes, we provide a domain-specific analysis, highlighting differential effects of rTMS on attention, executive functions, working memory, and episodic memory. This allows a more nuanced interpretation of treatment efficacy and better alignment with the multidimensional nature of PSCI.

Third, particular emphasis has been placed on the interaction between rTMS protocols and structured cognitive rehabilitation, an aspect that has received limited attention in prior reviews. Several studies suggest that combining neuromodulation with cognitive training may enhance cognitive outcomes compared with control conditions, supporting the hypothesis that rTMS may act as a facilitator of experience-dependent neuroplasticity. However, given that only a limited number of trials directly compare combined and single interventions, the evidence for a true synergistic effect remains preliminary.

Finally, this review provides a comparative overview of stimulation parameters and cortical targets, discussing differences between high-frequency rTMS, low-frequency stimulation, and iTBS, and their potential domain-specific effects. This contributes to identifying emerging trends and may inform the development of more standardized and personalized therapeutic protocols. Taken together, these elements extend previous knowledge by providing a more updated, methodologically rigorous, and clinically oriented synthesis of the evidence on rTMS for PSCI.

A crucial aspect that deserves consideration is the inter-individual variability in response to rTMS. While the present review primarily focuses on stimulation parameters and protocols, emerging evidence suggests that treatment outcomes may be significantly influenced by baseline patient characteristics. These include the severity and profile of cognitive impairment, time since stroke, lesion location, and network disruption, as well as neurophysiological and neuroimaging markers. Some studies included in this review provide indirect support for this perspective. For instance, changes in electrophysiological markers such as P300 latency reduction and amplitude increase have been reported following rTMS, suggesting enhanced cognitive processing efficiency and attentional resource allocation. Similarly, neuroimaging findings from resting-state functional magnetic resonance imaging (rs-fMRI) and functional near-infrared spectroscopy (fNIRS) indicate modulation of functional connectivity within fronto-parietal and default mode networks, including increased connectivity of the DLPFC cortex with cingulate, temporal, and parietal regions. In some studies, these changes correlated with improvements in cognitive performance, supporting a link between network reorganization and clinical outcomes [23,32,35].

As a whole, these findings suggest that rTMS may influence distributed cognitive networks rather than isolated cortical regions, promoting reorganization of large-scale connectivity patterns involved in attention, executive control, and memory processes. Nevertheless, the heterogeneity of methods and the limited number of studies incorporating multimodal biomarkers prevent definitive conclusions regarding causal mechanisms. Therefore, while the integration of biomarker data strengthens the biological plausibility of rTMS effects, further research is required to establish causal relationships between neurophysiological modulation and cognitive outcomes [35]. Notably, the neurophysiological and neuroimaging findings reported across several trials provide an important bridge between clinical outcomes and mechanistic interpretation, suggesting that observed cognitive improvements may reflect measurable changes in brain network dynamics rather than solely behavioral effects.

In addition, clinical factors, such as vascular risk profile or comorbid conditions (e.g., hypertension or depression), have been suggested to modulate responsiveness to stimulation [31,35]. These observations support a shift from a purely “protocol-centered” approach toward a more “patient-centered” and network-based framework, in which rTMS parameters are tailored according to individual neurobiological and clinical characteristics. In this scenario, the integration of biomarkers, such as neuroimaging connectivity patterns or electrophysiological indices, may help identify responders, optimize target selection, and guide treatment personalization. To date, however, evidence remains limited and largely exploratory, and most available studies are not specifically designed to assess predictors of response. Future research should incorporate stratification strategies and predictive modeling approaches to better define which patients are most likely to benefit from rTMS and to support the development of personalized rehabilitation protocols.

A preliminary aspect emerging from the present review is the geographical distribution of the included studies. The majority of RCTs identified were conducted in East Asian countries, particularly China and Taiwan, thus likely reflecting a strong research interest and rapid development of non-invasive brain stimulation techniques in these regions over the years considered here (2019–2025) [36]. However, evidence from other regions, including Europe and North America, supports the application of rTMS in post-stroke rehabilitation, although studies specifically targeting cognitive impairment remain comparatively fewer. In Western countries, indeed, rTMS has been more extensively investigated for motor recovery, depression, and aphasia [11,12,15,17], with growing but still limited research focusing on cognitive outcomes. The predominance of Asian studies in this review may therefore reflect differences in research priorities, study design focus, and publication trends rather than a true geographical restriction of rTMS use. Future multinational trials are warranted to improve generalizability, standardize protocols, and better define the role of rTMS in post-stroke cognitive rehabilitation across different healthcare systems.

**Figure 2:**
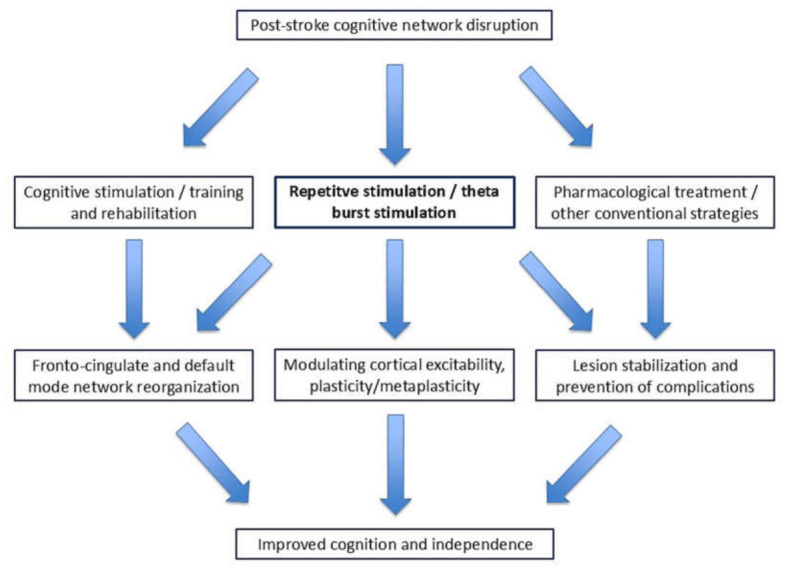
Conceptual model illustrating the mechanisms through which rTMS, including iTBS protocols, when combined with structured cognitive rehabilitation, may enhance network-level plasticity and promote cognitive and functional recovery after stroke.

A key element that emerged from the studies reviewed here is the role of integration between rTMS, including iTBS, and structured cognitive training. In almost all trials [22,23,24,30,32,33,34], stimulation was delivered in combination with computerized rehabilitation programmes or neurocognitive exercises, and the groups receiving both components showed greater gains [22,23,24,28,30] than those receiving training or stimulation alone, consistent with previous observations directly comparing rTMS + rehabilitation combinations with single interventions. This observation is compatible with the hypothesis that non-invasive neuromodulation may facilitate neuroplastic processes by enhancing the effects of traditional rehabilitation therapies and promoting the consolidation of skills practised during sessions [23,32,35]. Figure 2 illustrates the role of rTMS, including iTBS, within the main mechanisms hypothesized to take place in cognitive recovery after stroke.

Despite the consistency of the overall results, however, the available evidence presents some important heterogeneities that limit its strength. Protocols differ in frequency (1–20 Hz), intensity, number of pulses, cycle duration and, in part, cortical target [21,22,23,24,25,26,31,32,33,34,35], making it difficult to identify a single “gold standard” and hindering the possibility of a rigorous quantitative meta-analysis. This is particularly relevant because global cognitive screening tools, such as MMSE and MoCA, although useful for capturing overall clinical change, may show limited sensitivity to subtle deficits or domain-specific improvements and may be influenced by ceiling effects, especially in milder forms of PSCI. By contrast, studies using more specific instruments, such as extensive executive-function, attention, or memory test batteries, tended to provide a more nuanced profile of treatment effects, often showing stronger gains in attentional-executive domains rather than in global cognition alone. In addition, the sample size of many trials is relatively small and, critically for clinical translation, follow-ups are typically limited to immediate post-treatment assessments, and up to 4–12 weeks in the few studies only that included them. This precludes definitive conclusions about the durability of cognitive gains over months or years, despite partial maintenance observed in some trials with remote assessments.

A similar source of heterogeneity concerns the outcome measures used across studies. Most RCTs relied primarily on global cognitive screening tools such MMSE and MoCA, which were adopted as primary or secondary endpoints in the majority of included trials [22,23,24,25,28,29,30,32,35]. While these instruments are widely validated and clinically practical, they provide, as stated, a relatively coarse assessment of cognitive functioning and may not fully capture domain-specific changes. Although several studies complemented global scales with more detailed neuropsychological tests, such as the Trail Making Test, Digit Span Test, N-back tasks, and domain-specific batteries like the RBANS or LOTCA [22,23,24,26,31], this was not consistent across all trials. As a result, the apparent efficacy of rTMS may be influenced by the type and sensitivity of the outcome measures employed. This variability in outcome assessment may partly explain differences in reported effect sizes across studies and highlights the importance of using comprehensive and domain-specific neuropsychological batteries in future trials. More sensitive measures, possibly combined with neurophysiological (e.g., P300) or neuroimaging markers (e.g., functional connectivity changes on fMRI or fNIRS), as already explored in some studies [23,24,32,35], will provide a more accurate characterization of the cognitive effects of rTMS and help identify domain-specific treatment responses.

An additional relevant aspect concerns the role of lesion location in modulating the response to rTMS. PSCI is increasingly understood as a network-level disorder rather than the consequence of damage to a single anatomical region. However, most RCTs included in this review did not stratify patients according to lesion site, and specific evidence on location-dependent effects remains limited. Nevertheless, some preliminary observations suggest that rTMS may be effective even in patients with subcortical lesions, including thalamic stroke. For example, a case report described improvement in cognitive and behavioural symptoms following rTMS in a patient with thalamic infarction [37]. This finding supports the hypothesis that the effects of rTMS are mediated through the modulation of distributed cortico-subcortical networks rather than direct stimulation of the lesioned area. Accordingly, neurophysiological studies have shown that rTMS applied to cortical targets such as the DLPFC can induce changes in functional connectivity across widespread brain networks, potentially influencing subcortical structures, including the thalamus [10]. Given the central role of the thalamus in cognitive processes, its involvement in PSCI further supports the rationale for network-based neuromodulation approaches [38]. In this context, future studies should investigate whether lesion-specific or network-guided approaches (e.g., connectivity-based targeting) may improve treatment efficacy and allow more personalized neuromodulation protocols.

Finally, from a methodological point of view, the overall quality of the studies is good, but not without critical issues. Most trials have a low risk of bias in randomisation and outcome measurement, but in some cases the allocation procedure is not described in detail, the management of missing data is not clearly explained, or there are potential deviations from the planned intervention [21,22,23,24,25,26,31,32,33,34,35], especially in pilot studies. Furthermore, some studies use very small samples, designs that are not fully controlled (absence of a sham group or use of historical controls) or suboptimal sham TMS methods, limiting the generalisability of the results and increasing the risk of overestimating the effect. According to the GRADE assessment reported in our study, this translates into moderate to low certainty of evidence for several outcomes, particularly those related to specific cognitive domains and medium- to long-term effects. Importantly, the apparent convergence of findings in certain cognitive domains should not be interpreted as definitive evidence of reproducibility, as differences in study design, sample size, and assessment tools contribute to a heterogeneous evidence base that limits direct comparability.

### 4.2. Summary, Limitations, and Perspectives

The overall data suggest that rTMS may represent a potential adjunctive option in post-stroke cognitive rehabilitation. However, its clinical applicability remains limited at present due to several methodological constraints, including small sample sizes, short follow-up durations, and substantial heterogeneity in stimulation protocols and outcome measures. These factors reduce the generalizability of findings and prevent the definition of standardized treatment approaches suitable for routine clinical use [39,40,41].

The good tolerability emerging from the studies, with generally mild and transient adverse events [22,23,24,28,30,32] (such as headache, dizziness, local discomfort) and no serious event, supports its applicability even in fragile populations such as post-stroke patients, in line with reports in other neurological and psychiatric conditions [10,11,12,15,17]. However, considering the current evidence, rTMS cannot yet be considered a standard of care for PSCI, but rather a complementary intervention to be offered in expert centres and within controlled research settings, pending multicentre studies with larger samples, standardised protocols, and prolonged follow-ups (≥6–12 months). This will allow for a more precise definition of which stimulation patterns, durations, and combinations (e.g., with pro-cognitive drugs such as galantamine or donepezil) offer the best benefit/risk ratio in different patient profiles.

An additional critical aspect concerns the durability of the observed effects. Several studies reported improvements at short-term follow-up. However, follow-up duration was generally limited, often ranging from a few weeks to a maximum of three months. This makes it difficult to determine the long-term sustainability of rTMS-induced cognitive gains. This limitation has important implications for clinical translation, since cognitive rehabilitation aims not only to achieve short-term improvements but also to promote lasting functional recovery and independence. The absence of medium- to long-term follow-up data (e.g., ≥6–12 months) makes it difficult to determine whether the benefits of rTMS reflect transient modulation of cortical excitability or more stable neuroplastic changes. Furthermore, the lack of extended follow-up prevents evaluation of key clinically relevant outcomes, such as maintenance of cognitive gains, impact on daily functioning over time, and potential need for booster stimulation sessions. Future studies should incorporate longer follow-up periods and repeated outcome assessments to better define the persistence of treatment effects and to establish optimal maintenance strategies.

Lastly, although the review was conducted according to PRISMA guidelines and based on a structured PICO framework, the literature search was limited to two major databases (PubMed and Cochrane Library). While these sources ensure high-quality indexing of clinical trials, it is possible that relevant studies available in other databases, such as Scopus or Web of Science, were not captured. In addition, the search strings, particularly for PubMed, were designed to maintain specificity toward RCTs and clinically relevant outcomes, although this approach may have potentially excluded studies using alternative terminology for neuromodulation or cognitive impairment. Therefore, some degree of publication retrieval bias cannot be excluded. Future reviews will benefit from broader database inclusion and expanded search strategies to maximize coverage.

To summarize, the current evidence base does not yet support a routine clinical implementation of rTMS for PSCI. Most available trials are single-centre studies with relatively small cohorts and limited longitudinal assessment, often restricted to short-term outcomes. Moreover, the lack of standardized stimulation parameters and variability in cognitive assessment tools further complicate the translation of research findings into clinical protocols. These factors may increase the likelihood of overestimating treatment effects and limit the generalisability of the results. As a result, rather than indicating a ready-to-implement intervention, the available data should be viewed as an emerging research field requiring further validation through large-scale, multicentre trials with standardized methodologies. Future large-scale, rigorously designed trials are needed to confirm these findings and better quantify effect sizes across different clinical contexts.

## 5. Conclusions

Based on this systematic literature update, rTMS emerges as a potentially promising rehabilitation strategy for improving cognitive functions in patients with post-stroke deficits. While several studies suggest benefits, particularly in attentional-executive domains and working memory, the current evidence remains heterogeneous and does not yet support definitive conclusions regarding the consistency or generalizability of these effects. High-frequency protocols and iTBS applied to the left DLPFC show, in most trials, clinically relevant increases on neuropsychological scales and, in several cases, a favorable impact on activities of daily living. Nevertheless, the heterogeneity of protocols, the variety of assessment tools, the often-small sample sizes and, critically, the limited follow-up periods (rarely exceeding 3 months) reduce the overall certainty of the evidence and do not allow yet a “gold standard” stimulation pattern to be identified.

Multicenter studies with larger samples, shared protocols, prolonged follow-ups, and the integration of neurophysiological and neuroimaging biomarkers are needed to clarify the duration of the effects, the underlying mechanisms, and the patient profiles that benefit most. Considering the available data, rTMS should currently be regarded as an experimental or adjunctive intervention, primarily to be applied within specialized centers or research settings rather than as a standard component of routine clinical practice. This heterogeneity is also reflected in the results, where beneficial effects are not uniform across studies but appear to depend on several clinical and procedural variables.

Looking ahead, if further studies confirm the efficacy and safety of the most evidence-based protocols, rTMS will become a constant part of personalized post-stroke rehabilitation programmes, possibly in combination with intensive cognitive training and, in selected cases, with pro-cognitive/neuroprotective drugs. Future research should also focus on the personalization of rTMS protocols and on patients’ stratification according to their cognitive profiles (e.g., attention-executive vs. memory deficits), neuropsychiatric comorbidities (such as depression), and post-stroke phase (from hyperacute to chronic stage), as well as identify predictors of response and develop personalized, patient-centered neuromodulation strategies. Future studies should clarify efficacy, optimize protocols, and define responder profiles before routine clinical use can be recommended.

## Figures and Tables

**Table 1:** Summary of the main elements of the scientific articles considered for the systematic review.

N.	Authors (Year)	Disease Model (Stroke Phase)	Type of Study	Sample Size	Intervention	Control	Outcome	Follow-Up	Main Findings
1	Xu et al. (2024) [21]	PSCI (subacute/chronic; timing unspecified)	3-arm RCT, single-blind	48 patients (n = 15 dual-target, n = 15 single-target, n = 18 sham)	rTMS 10 Hz, 80% RMT for 4 weeks (5 sessions/week) on L-DLPFC + M1; 2000 pulses on L-DLPFC and 1200 on M1 in the double target group; real L-DLPFC + sham M1 in the single target group	Sham rTMS on L-DLPFC + M1 with coil tilted at 90° but same parameters, plus conventional rehabilitation in all groups	MoCA-BJ (primary outcome); MBI, TMT-A/B, DST (sequence and reverse sequence); serum levels of brain-derived neurotrophic factor and vascular endothelial growth factor	4 weeks (pre-treatment assessment and after 20 sessions)	Double-target rTMS significantly increased MoCA scores compared to sham and single target, reduced TMT-A times, and improved reverse DST in the active groups; brain-derived neurotrophic factor increased in both real groups, while vascular endothelial growth factor increased only in the double-target group and was higher than in the sham group.
2	Liu et al. (2020) [22]	Stroke outcomes with attention dysfunction (subacute)	Randomized, prospective, parallel, double-blind trial	58 patients (n = 29 active TMS, n = 29 sham), mean age ≈58 years	High-frequency TMS at 10 Hz on the left DLPFC (point F3), 90% RMT, 700 pulses per session, 5 days/week for 4 weeks, combined with comprehensive cognitive training on a touchscreen	Sham TMS with 90° coil at the same site and same parameters, plus the same cognitive training programme	FIM (motor, cognitive, total) for ADL; MMSE; TMT-A (time and errors); DST; Digit Span (forward and backward)	4 weeks (baseline and end-of-cycle assessment)	The TMS group showed significantly greater post-treatment gains in motor, cognitive and total FIM, MMSE, TMT-A performance (shorter time and fewer errors), DST, and Digit Span compared to sham, with no serious adverse events, indicating that 10 Hz TMS + cognitive training improves activities of daily living and attention after stroke.
3	Yin et al. (2020) [23]	PSCI (subacute)	Randomized clinical trial with rTMS group vs. control group without stimulation	34 PSCI patients: n = 16 rTMS, n = 18 control; fMRI subsample n = 14 (7 + 7)	10 Hz rTMS on L-DLPFC, 80% RMT, 2000 pulses per session (5 s × 40 trains, 25 s interval), once daily, 5 days per week for 4 weeks, followed by 30 min of computerized cognitive rehabilitation; standard drug therapy for all	Coil positioned perpendicular to the scalp (no-stim) at the same site, same session times, same cognitive rehabilitation, and drug therapy	MoCA (primary outcome); Stroop Test, times, and errors); RBMT; MBI; in fMRI subgroup: amplitude of low-frequency fluctuations and functional connectivity at rest	Assessments at baseline, after 2 weeks and after 4 weeks (end of 20 sessions)	rTMS resulted in significantly greater increases in MoCA, RBMT and MBI scores compared to the control group, with improvements also in Victoria Stroop Test times and errors, especially in the most complex conditions. These changes were associated with increased amplitude of low-frequency fluctuations in the left medial prefrontal cortex and greater functional connectivity between the left medial prefrontal cortex, right medial prefrontal cortex, and right ventral anterior cingulate cortex, correlating with cognitive and functional improvements.
4	Liu et al. (2024) [24]	Post-stroke working memory Deficits (subacute)	Randomised, parallel, double-blind, sham-controlled trial	100 patients with stroke and working memory deficits randomized (50 rTMS, 50 sham); 82 completed the trial	10 Hz rTMS on the left DLPFC localized with MRI neuronavigation; 90% RMT, 1280 pulses per session, 18 min, 1 session/day for 14 consecutive days (2 weeks)	Sham rTMS with placebo coil identical in appearance, sound, and vibration but without effective magnetic field, same parameters and duration	Primary: accuracy in the N back visual test Secondary: MMSE, MoCA, ADL, IADL, DST, Wisconsin Card Sorting Test, Symbol Digit Modalities Test, fNIRS (oxygenated hemoglobin and FC in DLPFC, PMC, superior parietal lobule), adverse events.	Assessments at baseline (T0), at the end of the intervention (week 2) and 4 weeks after completion (week 6)	The rTMS group showed significantly greater improvements in all levels of the N back test at 2 and 6 weeks compared to sham, with partial maintenance of the effect at 6 weeks; MMSE, MoCA, IADL, attention and executive function (DST, Wisconsin Card Sorting Test, Symbol Digit Modalities Test) also improved, along with an increase in oxygenated hemoglobin and connectivity between the left DLPFC, right PMC and right superior parietal lobule; side effects (mainly mild headache) were frequent but well tolerated and similar between groups.
5	Li et al. (2022) [25]	PSCI (subacute)	Prospective, single-centre, randomised, double-blind, pseudo-controlled RCT	60 PSCI patients enrolled; n = 30 assigned to i or iTBS, n = 30 to sham; 58 completed the study (28 iTBS, 30 sham)	iTBS on the left DLPFC (point F3, 10–20 system); intensity 100% RMT; triplets at 50 Hz repeated at 5 Hz, pattern 2 s on/8 s off, total 600 pulses in 192 s; 1 session/day, 5 days/week for 2 weeks, combined with donepezil 10 mg/day and standard cognitive training	Sham iTBS with coil rotated 90° on the same area (minimum stimulation), same parameters and same pharmacological l and cognitive rehabilitation	MMSE (global cognition); Oxford Cognitive Screen (modules: picture naming, semantics, orientation, visual field, sentence reading, number/calculation, imitation, memory, executive task); ERP P300 (latency and amplitude)	Baseline assessments (T0) and immediately after 10 sessions (T1, end of 2 weeks); no long-term follow-up	Both groups showed improvements, but iTBS produced significantly greater increases in MMSE, more marked improvement in semantics and executive tasks on the Oxford Cognitive Screen, and a greater reduction in P300 latency with increased amplitude, indicating a favorable effect on global cognition, executive functions, and processing speed; no serious adverse events, only a transient episode of sneezing
6	Chu et al. (2022) [26]	PSCI (subacute/chronic; unspecified)	Prospective, randomised, single-blind, 3-arm controlled study (iTBS + training, tDCS + training, cognitive training alone)	60 PSCI patients: n = 21 iTBS, n = 19 tDCS, n = 20 control; 30 sessions over 6 weeks for all	iTBS on left DLPFC (F3, 10–20 system): 70% RMT, triplets at 50 Hz repeated at 5 Hz, 2 s on/8 s off, 600 pulses in 3 min 20 s, 1 session/day for 30 weekdays; after each session, computer-assisted cognitive rehabilitation (attention, executive function, memory, calculation, reasoning) 30 min	tDCS: anode on left DLPFC (F3), cathode on contralateral shoulder, 2 mA for 20 min, 30 sessions + same cognitive training; control group: cognitive training only (30 min, 5 ×/week for 6 weeks)	Primary: LOTCA (total and subscales: orientation, visuospatial perception, visuomotor organization, operational thinking, attention); Secondary: MBI for ADL; subgroup (n = 7 iTBS, n = 7 tDCS) undergoing fNIRS during verbal fluency test for Oxygenated hemoglobin and prefrontal activation patterns	Assessments at baseline and at the end of the 6-week treatment period; no long-term follow-up	All groups improved their LOTCA scores, but the increase was significantly greater with iTBS + training and tDCS + training than with training alone, particularly for visuomotor organization and operational thinking, and for attention only in the iTBS group. MBI increased significantly in the iTBS and tDCS groups but not in the control group, and iTBS/tDCS showed higher final MBI scores; at fNIRS, iTBS activated the left DLPFC, frontopolar cortex and Broca’s area, while tDCS mainly activated the frontopolar cortex, suggesting partially different mechanisms of cognitive improvement.
7	Hu et al. (2023) [27]	Post-stroke memory Deficit (chronic)	Preliminary randomized 3-arm study (sham, rTMS, rTMS + tDCS) with blinded assessor	34 patients with postictal memory deficit: n = 12 sham, n = 12 rTMS, n = 10 rTMS + tDCS	rTMS: 5 Hz on left DLPFC (F3), 80% RMT, 1200 pulses/day (5 s trains with 25 s pauses), 20 min, 5 days/week for 4 weeks; in the combined	Sham group: same cognitive rehabilitation but without rTMS or tDCS; all groups received computerized cognitive training focused on memory, motor rehabilitation, and ADL. rTMS + tDCS group, rTMS as above + simultaneous tDCS with anode on the affected temporal lobe (T5/T6) and cathode on the contralateral posterior parietal cortex (P3/P4), 1.2 mA for 20 min	Total MoCA and sub-items (in particular, delayed recall); total RBMT 3 and individual daily memory tests; mismatch negativity ERP (latency) and P300 ERP (latency and amplitude) recorded at Cz.	Assessments on the day before the start (PRE) and the day after the end of the 4-week treatment (POST); no long-term follow-up	All groups improved their MoCA scores, but the rTMS and, above all, rTMS + tDCS groups showed greater increases and a more marked improvement in delayed recall; in the RBMT, several items (belongings, orientation, recall of stories and routes, in particular delayed route recall) improved only in the active groups, with a significant advantage for the combination compared to rTMS alone; mismatch negativity and P300 latencies shortened and P300 amplitude increased in the active groups, with more pronounced changes in the rTMS + tDCS group, and these changes were correlated with RBMT scores, indicating that bimodal stimulation is more effective than cognitive rehabilitation alone or rTMS alone for post-stroke iTBS amnesia.
8	Li et al. (2021) [28]	Cognitive impairment in the post-stroke recovery phase (subacute)	Single-centre, parallel-group RCT, rTMS vs. sham, with conventional rehabilitation in both groups	70 randomised patients (35 rTMS, 35 sham); final analysis on 65 patients: n = 33 rTMS, n = 32 sham	Low frequency (1 Hz) rTMS on the contralateral DLPFC (F3/F4, 10–20 system), intensity 90% MT, 1000 pulses in 20 min (10 s trains with 3 s pauses), 1 session/day, 5 days/week for 4 weeks (20 sessions) + standard medical therapy, motor rehabilitation, and structured cognitive training 30 min/day	Sham rTMS on the same area with coil rotated 90° (ineffective field), same time parameters and same pharmacological, motor, and cognitive rehabilitation	MMSE, MoCA total and subdomains (visuospatial, memory, language, attention), MBI for ADL; thyroid hormones and TSH; correlation and regression analysis between hormones and MoCA	Assessments one day before the start and one day after the end of the 4-week treatment; no long-term follow-up	At baseline, T3, FT3, and TSH were positively correlated with MoCA scores, while thyroid hormones were not; after 4 weeks, both groups improved MoCA, MBI, and cognitive domains, but the increase in MoCA, MBI, and the visuospatial, memory, and attention subscales was significantly greater in the rTMS group; rTMS resulted in a more marked increase in thyroid hormones and TSH compared to sham, and in rTMS patients, increases in thyroid hormones were associated with gains in MoCA and specific domains (visuospatial, memory, attention), suggesting a possible role mediated by the hypothalamic-pituitary-thyroid axis in post-stroke cognitive modulation.
9	Song et al. (2025) [29]	Cognitive impairment in the subacute post-stroke phase (subacute)	Randomised study, real vs. sham rTMS, with extensive neuropsychological, neurophysiological, and functional connectivity assessment	28 patients with first stroke and cognitive deficits, assigned to real or sham rTMS; 10 sessions over 2 weeks	High-frequency 10 Hz neuronavigated rTMS on the left DLPFC (80% rMT, 5 s trains with 25 s pauses, 1500 pulses per session), for 10 sessions over 2 weeks, in addition to standard rehabilitation	Sham rTMS with the same procedure but without effective magnetic field output, plus the same standard rehabilitation	Clinical scales (Korean version): MMSE, MoCA, ADL, EQ-5D (QoL), GDS (depression), Continuous Performance Test; and Vascular Cognitive Impairment Harmonization Standards (executive functions/memory); neurophysiology: motor evoked potential and ERP (P300); neuroimaging: rs fMRI and diffusion tensor imaging (cingulate).	Assessments at baseline, 1 month and 3 months after stroke; neurophysiology and neuroimaging at baseline and 3 months	The rTMS group showed significantly greater improvements in MMSE, MoCA, MBI, and GDS compared to sham, with the benefit on MoCA maintained at 3 months; vascular cognitive impairment Harmonization Standards Z scores for executive functions and memory increased more in the rTMS group; neurophysiologically, an increase in intracortical inhibition and fronto-central P300 amplitude was observed, while rs fMRI showed increased connectivity in the cingulate, supramarginal gyrus, cerebellar crus II, precentral and temporal areas; changes in MoCA positively correlated with the anisotropy fraction of the cingulate, suggesting that cognitive improvement is mediated by modulation of the fronto-cingulate networks.
10	Hu et al. (2024) [30]	Cognitive impairment/v vascular dementia after first ischemic stroke (within 6 months) (subacute)	Three-arm RCT: rTMS + cognitive rehabilitation vs. galantamine + cognitive rehabilitation vs. rTMS + galantamine + cognitive rehabilitation; no sham/placebo group for ethical reasons	90 patients (30 per group), aged 50–80 years, first ischaemic stroke; all diagnosed with vascular dementia according to 2016 Chinese guidelines	high-frequency (5 Hz) rTMS on the left DLPFC (80% MT, 3000 pulses/day), 1 session/day, 5×/week for 4 weeks, plus structured cognitive rehabilitation, standard medical therapy, and motor rehabilitation	Oral galantamine (second generation, acetylcholinesterase inhibitor) + same cognitive and motor rehabilitation; combined group: rTMS 5 Hz + galantamine + cognitive and motor rehabilitation according to the same protocols	MMSE, MoCA (8 cognitive domains), Fugl Meyer motor, modified Barthel index for ADL; serum markers: homocysteine and neuron-specific enolase as indices of neuronal damage	Assessments before initiation and after 4 weeks of treatment; no long-term follow-up reported	All three groups showed significant improvements in MMSE, MoCA, Fugl Meyer, and Barthel scores and a reduction in neuron-specific enolase and homocysteine compared to baseline, but the combined rTMS + galantamine + cognitive rehabilitation group achieved the highest cognitive and motor scores and the greatest reduction in homocysteine and neuron-specific enolase, with significant differences compared to the rTMS-only and galantamine-only groups. The authors hypothesized a synergistic effect through more extensive activation of the cholinergic system and modulation of synaptic plasticity and neurovascular inflammation.
11	Tsai et al. (2020) [31]	PSCI after left hemispheric stroke (>3 months) (subacute/chronic))	Randomised, controlled, double-blind, 3-arm RCT (5 Hz rTMS vs. iTBS vs. sham)	44 enrolled, assigned to 5 Hz rTMS (n = 14), iTBS (n = 15), sham (n = 15); final analysis: 5 Hz n = 11 (3 dropouts), iTBS n = 15, sham n = 15	44 enrolled, assigned to 5 Hz rTMS (n = 14), iTBS (n = 15), sham (n = 15); final analysis: 5 Hz n = 11 (3 dropouts), iTBS n = 15, sham n = 15	iTBS: burst of 3 pulses at 50 Hz repeated at 5 Hz, 2 s on/8 s off, 600 total pulses in 190 s, intensity 80% RMT, same number of sessions; sham: same procedure and positioning with placebo coil (<5% output)	RBANS battery (total and indices: immediate memory, visuospatial/constructive, language, attention, delayed memory) and Beck Depression Inventory, assessed at baseline and 1 day after the 10th session	No long-term follow-up; assessment only before and immediately after treatment	Both active groups (5 Hz and iTBS) showed significant increases in total RBANS scores compared to baseline, while sham remained unchanged; compared to sham, both 5 Hz and iTBS resulted in greater improvement in total RBANS; 5 Hz rTMS markedly improved attention and delayed memory and was superior to iTBS in the attention domain, while iTBS mainly improved immediate memory, language and delayed memory; depression did not change significantly; Patients without hypertension appeared to respond better to treatment, suggesting that both 5 Hz rTMS and iTBS on the left DLPFC are effective for PSCI, with a possible advantage of 5 Hz on attention.
12	Li et al. (2020) [32]	Cognitive impairment after first haemorrhagic stroke (basal ganglia/corona radiata) (subacute)	Prospective, single-centre, randomised, double-blind, sham-controlled study with resting fMRI	30 patients with haemorrhagic stroke SCI: n = 15 rTMS, n = 15 control; all within 3 months of the event, age 50–75 years	High-frequency 5 Hz rTMS on the left DLPFC (F3, 10–20), 100% MT, 50 trains of 40 pulses (2000 pulses/session) with 25 s interval, 20 min/day, 5×/week for 3 weeks (15 sessions) + structured multimodal cognitive training 30 min/day	Sham rTMS with the same procedure but with the coil perpendicular to the scalp (ineffective field), same cognitive training for 3 weeks	MMSE and MoCA (Chinese version) for global cognitive function and domains; rs fMRI before and after 3 weeks for fractional amplitude of low-frequency fluctuations (local spontaneous activity) and seed-based functional connectivity from the left DLPFC	Clinical assessments and fMRI at baseline and after 3 weeks of treatment; no follow-up beyond the intervention period	Both groups improved on the MMSE and MoCA, but the rTMS group had a significantly greater cognitive increase; at rs-fMRI, rTMS increased fractional amplitude of low-frequency fluctuations in the superior temporal gyrus, inferior frontal gyrus, and para-hippocampal gyrus and reduced it in the middle temporal gyrus, middle frontal gyrus and fusiform gyrus; DLPFC-precuneus, DLPFC-middle/inferior frontal, DLPFC-inferior temporal gyrus, and DLPFC-marginal gyrus connectivity increased, while DLPFC-middle temporal gyrus and DLPFC-thalamus connectivity decreased. The increase in FC between DLPFC and precuneus/frontal/marginal correlated with MoCA improvement, indicating that cognitive benefit was mediated by a reorganization of fronto-temporal and default mode networks.
13	Cha et al. (2022) [33]	PSCI in the chronic phase, with post-stroke depression (chronic)	Prospective pilot study, single arm with historical control group; high frequency rTMS on ipsilesional DLPFC, 10 sessions	10 PSCI patients (6 ischemic strokes, 4 hemorrhagic strokes), PSCI duration ≥6 months (mean ~30 months), all with depression (GeDS ≥10)	High-frequency (20 Hz) rTMS on ipsilesional DLPFC: 100% RMT, 5-s trains with 55-s intervals for 20 min (2000 pulses/session), 5×/week for 2 weeks (10 sessions total); no change in usual rehabilitation, instructions for homebased cognitive training	No parallel sham group; comparison with 11 chronic PSCI patients without rTMS, with repeated MMSE after ~14 months	Cognitive tests: MMSE, MoCA, Wechsler Adult Intelligence Scale-IV, auditory verbal learning test, Complex Figure Test, Memory Quotient, global GDS, CDR-SB, motor scales (Berg Balance Scale, Manual Function Test; Fugl Meyer), ADL (MBI, IADL), QoL (Stroke Specific QoL); biomarkers: mRNA IL-6, IL-1beta, tumor necrosis factor-alpha, Transforming Growth Factor beta and C-reactive protein; cognitive fMRI on 2 patients	Assessments at baseline, 2 weeks (end of rTMS) and 14 weeks; historical control with MMSE at baseline and ~14 months	After 10 sessions, IQ, Memory Quotient, auditory verbal learning test, CFT, QoL and Manual Function Test improved significantly; at 14 weeks, maintenance/further improvement in auditory verbal learning test, CFT and Memory Quotient was observed, along with an increase in MMSE and MoCA and a reduction in CDR-SB, with late motor improvements (Berg Balance Scale, Trunk Impairment Scale); the historical group showed no significant changes in MMSE, while rTMS patients did; pro-inflammatory cytokines (IL-6, IL-1beta, tumor necrosis factor-alpha, transforming growth factor beta were reduced immediately after rTMS, with Interleukin-1 beta still low at 3 months, and the reduction in IL-6 strongly correlated with gains in auditory verbal learning test and CFT; fMRI in 2 patients showed greater post-rTMS activation in areas related to language, memory and executive control (angular gyrus, medial frontal cortex, hippocampus), suggesting that high-frequency rTMS on the ipsilesional DLPFC may induce lasting cognitive improvement mediated by anti-inflammatory responses and network reorganization.
14	Yingli et al. (2020) [34]	PSCI within 6 months of stroke (ischaemic or hemorrhagic) (subacute))	Prospective, single-centre, double-arm (1 Hz rTMS vs. sham) RCT on a background of conventional cognitive training	36 patients with PSCI, aged 38–75 years: n = 18 rTMS, n = 18 control; groups balanced for age, sex, stroke type, and side of lesion (predominantly left hemisphere)	Low- frequency 1 Hz rTMS on the DLPFC of the unaffected hemisphere (F3 or F4, 10–20 system), 80% MT, 30 sequences of 20 pulses (600 pulses/session ), 1 session/day, 5×/week for 8 weeks, combined with structured cognitive rehabilitation 30 min/day	Sham rTMS with same parameters and positioning but Coil perpendicular to the skull (ineffective field), same cognitive rehabilitation, and basic medical therapy	LOTCA (global cognitive functions and executive, visuospatial, attention subdomains, etc.); ERP P300 (latency and amplitude) as a neurophysiological index of cognitive processing; assessed pre- and post-8 weeks	Measurements at baseline and after 8 weeks of treatment; no long-term follow-up is planned.	After 8 weeks, both groups showed an increase in LOTCA scores and a shortening of P300 latency with increased amplitude, but the improvements were significantly greater in the rTMS group than in the sham group. The authors conclude that 1 Hz rTMS on the contralateral DLPFC, added to cognitive training, can enhance cognitive recovery in PSCI, probably by modulating cortical excitability and LTP-like plasticity of cognitive circuits.
15	Li et al. (2024) [35]	PSCCID: cognitive impairment + depression after first stroke (within 12 weeks) (subacute)	Prospective, single-centre, randomised, double-blind, controlled RCT (rTMS vs. sham) with rs-fMRI and P300	30 PSCCID patients, aged 45–75, righthanded: n = 15 rTMS, n = 15 sham, groups balanced for age, stroke type and side, duration, education	High-frequency (10 Hz) rTMS on the left DLPFC (8 coil): 100% RMT, 39 trains of 30 pulses (1170 pulses/session) with 28 s intervals, 20 min/session, 5×/week for 4 weeks (20 sessions) + standard drug therapy (including sertraline 50 mg/day) and conventional motor, cognitive, psychological rehabilitation	Sham rTMS with the same procedure but coil perpendicular to the skull (ineffective field) + same medical therapy (including sertraline) and standard rehabilitation	Cognition: MMSE; depression: HDRS-17; neurophysiology: P300 (amplitude and latency at Pz); rs fMRI (functional connectivity analysis within the default mode network) pre- and post-cycle	Clinical assessments, P300 and rs fMRI at baseline and after 4 weeks of treatment; no follow-up beyond immediate post-intervention	Both groups showed improvements in MMSE, HDRS-17 and P300 parameters, but the rTMS group had significantly greater increases in both cognition and depression compared to sham; in the default mode network, rTMS increased connectivity between the left temporal pole/left parahippocampus and right lateral temporal cortex/right retrosplenial cortex, with these FC indices positively correlated with MMSE scores and some P300 characteristics; The authors conclude that 10 Hz stimulation of the left DLPFC is effective in simultaneously improving cognitive deficits and depressive symptoms in PSCCID, probably through compensatory remodeling of connections within the default mode network.

Legend (in alphabetical order): ADL, Activities of Daily Living; CDR-SB, Clinical Dementia Rating Scale-Sum of Boxes; CFT, Complex Figure Test; DLPFC, dorsolateral prefrontal cortex; DST, Digit Span Test; EQ-5D-EuroQol, 5-Dimensions Questionnaire (Quality of Life); ERP, event-related potentials; FC, functional connectivity; FIM, Functional Independence Measure; fMRI, functional magnetic resonance imaging; fNIRS, functional near-infrared spectroscopy; GDS, Geriatric Depression Scale; HDRS-17, 17-item Hamilton Depression Rating Scale; IADL, Instrumental Activities of Daily Living; IL, interleukin; iTBS, intermittent theta burst stimulation; LOTCA, Loewenstein Occupational Therapy Cognitive Assessment; MBI, Modified Barthel Index; MMSE, Mini-Mental State Examination; MoCA, Montreal Cognitive Assessment; MT/RMT, (resting) motor threshold; P300, P300 component of event-related potentials; PMC, premotor cortex; PSCI, post-stroke cognitive impairment; PSCCID, post-stroke cognitive impairment with co-existing depression; QoL, quality of life; RBANS, Repeatable Battery for the Assessment of Neuropsychological Status; RBMT, Rivermead Behavioural Memory Test; RCT, randomized controlled trial; rTMS, repetitive transcranial magnetic stimulation; rs-fMRI, resting-state functional magnetic resonance imaging; SCI, stroke-related cognitive impairment; tDCS, transcranial direct current stimulation; TMT-A/B, Trail Making Test A/B; TSH, thyroid-stimulating hormone; stroke phase classification was derived from study-reported time since stroke and categorized as acute (≤1 month), subacute (1–6 months), and chronic (>6 months). When not explicitly specified, classification was inferred from inclusion criteria.

**Table 2:** Summary of the risk of bias assessments for each study.

N.	Authors (Year)	Randomisation	Intervention Deviations	Missing Data	Outcome Measurement	Outcome Selection	Overall Risk
1	Xu et al. (2024) [21]	Moderate	Low	Moderate	Low	Low	Moderate
2	Liu et al. (2020) [22]	Low	Low	Low	Low	Low	Low
3	Yin et al. (2020) [23]	Low	Low	Low	Low	Low	Low
4	Liu et al. (2024) [24]	Low	Low	Low	Low	Low	Low
5	Li et al. (2022) [25]	Low	Low	Low	Low	Low	Low
6	Chu et al. (2022) [26]	Low	Low	Low	Moderate	Low	Moderate
7	Hu et al. (2023) [27]	Moderate	Moderate	Low	Moderate	Low	Moderate
8	Li et al. (2021) [28]	Moderate	Low	Low	Low	Low	Low
9	Song et al. (2025) [29]	Moderate	Moderate	Low	Moderate	Moderate	Moderate
10	Hu et al. (2024) [30]	Moderate	Low	Low	Moderate	Low	Moderate
11	Tsai et al. (2020) [31]	Low	Low	Low	Low	Low	Low
12	Li et al. (2020) [32]	Low	Low	Low	Low	Low	Low
13	Cha et al. (2022) [33]	High	High	Low	Moderate	Low	High
14	Yingli et al. (2022) [34]	Moderate	Low	Low	Moderate	Low	Moderate
15	Li et al. (2024) [35]	Low	Low	Low	Low	Low	Low

## Data Availability

All relevant data are included in the manuscript and its Supplementary Materials.

## Supplementary Materials

The following supporting information can be downloaded at: https://www.mdpi.com/article/10.3390/life16040700/s1, Table S1: PRISMA 2020 Checklist. Reference [42] is cited in the supplementary materials.
